# Comparing Conventional and Supercritical Extraction of (−)-Mammea A/BB and the Antioxidant Activity of *Calophyllum brasiliense *Extracts

**DOI:** 10.3390/molecules18066215

**Published:** 2013-05-24

**Authors:** Renata Menoci Gonçalves, Caroline Ortega Terra Lemos, Ivana Correa Ramos Leal, Celso Vataru Nakamura, Diógenes Aparício Garcia Cortez, Edson Antonio da Silva, Vladimir Ferreira Cabral, Lúcio Cardozo-Filho

**Affiliations:** 1 Programa de Pós-Graduação em Engenharia Química, Universidade Estadual de Maringá (UEM), Av. Colombo, 5790, Bloco E-46, 87020-900 Maringá, Paraná, Brazil; 2 Faculdade de Farmácia, Departamento de Produtos Naturais e Alimentos, Universidade Federal do Rio de Janeiro, Av. Carlos Chagas Filho, 373, Bloco A, 2°andar, Ilha do Fundão, Cidade Universitária, 21941-902, RJ, Rio de Janeiro, Brazil; 3 Programa de Pós-Graduação em Ciências Farmacêuticas, Av. Colombo, 5790, Bloco K-68, 87020-900 Maringá, Paraná, Brazil; 4 Programa de Pós-Graduação em Engenharia Química, Universidade Estadual do Oeste do Paraná (UNIOESTE), Caixa postal 520, 85903-000,Toledo, Paraná, Brazil

**Keywords:** *Calophyllum brasiliense*, supercritical extraction, (−)-mammea A/BB, antioxidant activity, total phenols, mathematical modeling

## Abstract

*Calophyllum brasiliense* is a rich source of bioactive coumarins, xanthones and biflavonoids. The aim of the study was to compare the phenol contents and the antioxidant activity of *C. brasiliense* extracts obtained by conventional and supercritical fluid extraction (SFE) methods, as well as the quantification of crude extracts and (−)-mammea A/BB yields. Dichloromethane and hexane were used as solvents for the conventional extractions and SFE was developed using supercritical CO_2_; the kinetic curves were modeled using a second-order empirical model. The dichloromethane extract presented the best total yield, although it showed the lowest content of (−)-mammea A/BB. The concentration of the coumarin was considerably higher in extracts obtained by the supercritical fluid method and a higher antioxidant activity was assigned to extracts obtained by this technique. Concerning the total phenolic contents, both the dichloro-methane and the supercritical extractions produced satisfactory amounts. The SFE method proved to be more promising than conventional methods.

## 1. Introduction

The genus *Calophyllum* (Clusiaceae/Guttiferae) comprises an extensive group of tropical trees with approximately 180–200 species restricted to the hot and humid tropics [1]. The genus includes various trees, shrubs, lianas and herbs of economical interest for the production of fruits, timber, chemical compounds with pharmaceutical properties and paints [2].

*Calophyllum brasiliense C*ambess, popularly known as *guanandi* [3], is a rich source of bioactive compounds such as coumarins, xanthones, steroids, triterpenes and bioflavonoids [4,5,6,7,8]. Ethnopharmacological studies have already reported the use of this species against bronchitis, gastritis, hepatitis [9], pain [10], inflammations, diabetes, hypertension [11], diarrhea [12] and herpes [13]. It is one of the most studied species due to its biological activities, with special attention to the antibacterial [14,15,16], antifungal [14], cytotoxic [16], tumor inhibitory [17], and HIV-1 IIIb/LAV replication inhibitory, which are attributed to the leaves, stems and roots extracts [18]. Extracts and fractions of its leaves have demonstrated leishmanicidal effects against promastigotes and amastigotes of *Leishmania amazonensis* [19,20], as well as antiviral activity [21].

Traditionally, the extraction of bioactive compounds from herbs has been performed by steam distillation or by the use of organic solvent-based methods such as the maceration, percolation and Soxhlet techniques. An alternative method is the use of the supercritical fluid technology that employs gases above their critical pressures and temperatures as solvents to selectively extract soluble components from raw materials [22]. Carbon dioxide (CO_2_) has gained the best acceptance since it offers many advantages, such as mild supercritical conditions, low cost, easy manufacture, non-toxic and non-flammable properties, ready availability and easy removal from the extracted products [23]. Beside this, the use of carbon dioxide provides the advantage of being suitable for extracting thermo- labile compounds due the fact that excessive solvent heating is not necessary [24,25]. Nowadays, supercritical fluid extraction, which was developed in 1960, is used in a wide variety of areas, including the ood, pharmacy and environmental engineering industries [26].

Within this context, the objective of the present work was to study the chemical composition and the biological activity of leaf extracts from *Calophyllum brasiliense* Cambess, obtained by conventional and SFE methods. The experiments with supercritical CO_2_ were carried out in a laboratory scale unit at different temperature and pressures, but at a constant solvent flow rate. Two different granulometries of the vegetal samples were also considered (30 and 50 mesh). Selected extracts obtained by conventional and SFE methods were further subjected to antioxidant activities and phenolic compounds assays. These extracts were also analyzed by high performance liquid chromatography (HPLC) for their (−)-mammea A/BB contents since it has important biological activity, mainly against protozoans and tumors [27], high cytotoxic activity against some tumor cell lines [16,28], molluscicidal activity against the *Biomphalaria glabrata*s nail [29] antileishmanial activity against *L. amazonensis* [19,20] and trypanocidal effects in vitro against *Trypanosoma cruz* [30]. The kinetic curves of the extraction were correlated by a second-order empirical model.

## 2. Results and Discussion

### 2.1. Overall Yield of Extraction

Table 1 shows the average values for the total yields obtained by the supercritical fluid and organic solvent extraction methods. The results indicate that the extractions using organic solvents (dichloromethane and hexane) produced higher total yields than those obtained with carbon dioxide. The highest yields were obtained with dichloromethane, which is a polar solvent. This behavior can be attributed to the higher temperature, solvent recirculation and solute-solvent interactions found in the Soxhlet extraction method [31].

**Table 1:** Overall yields of *C. brasiliense* extracts obtained by SFE and Soxhlet methods.

Supercritical fluid extraction					Soxhlet extraction	
Operational conditions			Yield (%) ^a^		Solvent	Yield (%) ^a^
T (K)	Pressure (MPa)	CO_2_ density (g/cm³)	Mesh 30	Mesh 50		
313	10.92	0.6813	1.4 ± 0.02	1.6 ± 0.01		
313	15.00	0.7811	2.0 ± 0.06	2.2 ± 0.04	Dichloromethane	4.3 ± 0.03
333	17.67	0.6813	2.2 ± 0.04	2.3 ± 0.05	Hexane	3.9 ± 0.01
333	24.41	0.7811	2.9 ± 0.02	2.9 ± 0.06		
313	25.00	0.8802	2.8 ± 0.02	2.8 ± 0.04		

^a^ Yield (%) = (extracted mass/mass of dry sample) × 100.

The two main factors affecting SFE are pressure and temperature. Increasing pressure at constant temperatures raises the density and the solvating capacity of the supercritical CO_2_. In fact, as can be seen in Table 1, higher extraction yields are observed with increasing pressure at constant temperature (10.9; 15 and 25 MPa at 313K) (17.6 and 24.4 at 333K), for both meshes tested. It can also be noticed that at a constant density, an increase in the extraction yields was accomplished when the temperature and pressure were increased. This behavior can be clearly observed in this investigation by comparing the yield obtained at 10.92 MPa with that at 17.67 MPa and, at 15.00 MPa with that at 24.41 MPa. These results are in agreement with data previously reported in the literature [32,33].

The solvent power of a supercritical fluid can also be directly related to its fluid density, so it is not strange that the extraction yield is enhanced when the density increases. An increase in temperature reduces the density of the solvent, thus reducing solubility, then when the density is decreased from 0.7811 to 0.6813 g/cm³, at the same temperature, the yield of extraction is decreased. This process is better explained by a balance between density-solvent power and vapor pressure of the solute.

**Figure 1:**
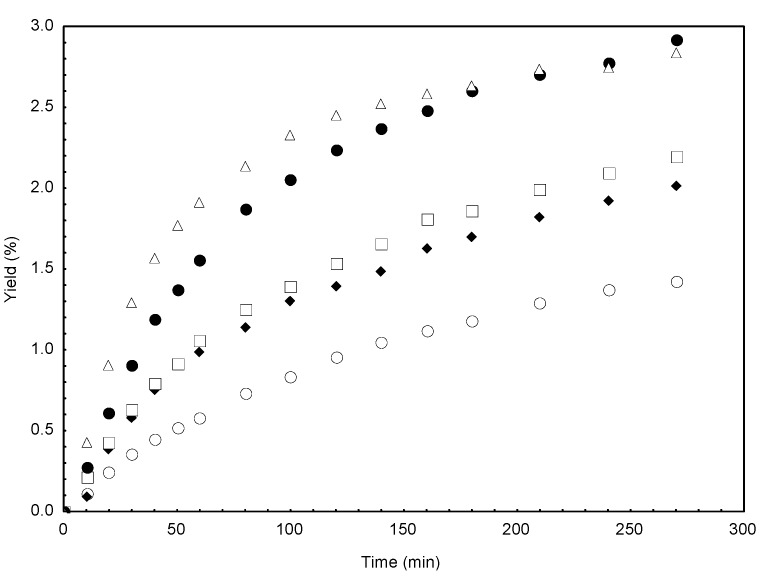
Supercritical fluid extraction curves of *Calophyllum brasiliense* leaves by mesh 30. (○) P = 10.92 MPa, T = 313 K, ρ = 0.6813 g/mL; (♦) P = 15.00 MPa, T = 313 K, ρ = 0.7811 g/mL; (□) P = 17.67 MPa, T = 333 K, ρ = 0.6813 g/mL; (●) P = 24.41 MPa, T = 333 K, ρ = 0.7811 g/mL; (∆) P = 25.00 MPa, T = 313 k, ρ = 0.8802 g/mL.

**Figure 2:**
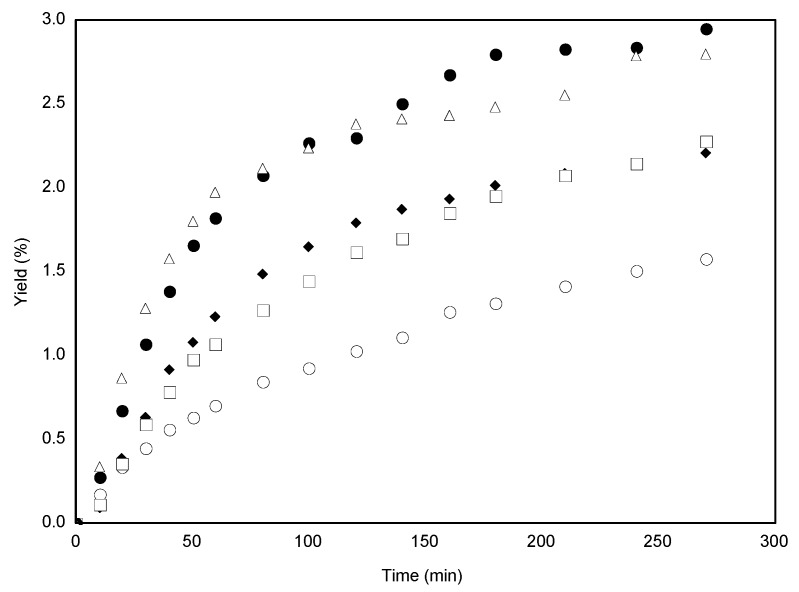
Supercritical fluid extraction curves of *Calophyllum brasiliense *leaves by mesh 50. (○) P = 10.92 MPa, T = 313 K, ρ = 0.6813 g/mL; (♦) P = 15.00 MPa, T = 313 K, ρ = 0.7811 g/mL; (□) P = 17.67 MPa, T = 333 K, ρ = 0.6813 g/mL; (●) P = 24.41 MPa, T = 333 K, ρ = 0.7811 g/mL; (∆) P = 25.00 MPa, T = 313 k, ρ = 0.8802 g/mL.

Figure 1 and Figure 2 illustrate the CO_2_ supercritical fluid extraction kinetic curves for *C. brasiliense* leaves of 30 and 50 mesh, respectively. Both figures show that independently of the granulometry, a time-dependent yield increase is observed. In Figure 1 it is observed that, until 220 min, as the CO_2_ density and pressure are increased a higher yield is obtained. Therefore, at the last 50 minutes a decrease in the yield was observed even under high pressure and CO_2_ density conditions. This can be explained considering that for longer time periods, solubility is the dominant factor for overall yield. Since the extract is composed by different classes of compounds, their solubility may also be different for the various temperature and pressure conditions.

As could be seen, Table 1 reveals that under the same conditions, there was no significant difference at 5% (Tukey’s test) for overall yield between the different granulometry. In addition, Figure 1 and Figure 2 show that, independent of the granulometry, the yield amount is time-dependent. Therefore, mesh 30 particles were selected for the antioxidant tests, for the mathematical modeling and quantification of the compound (−)-mammea A/BB.

### 2.2. (-) Mammea A/BB Content

The extracts showed different chromatographic profiles and concentrations of the compound (−)-mammea A/BB. Table 2 shows the mass percentage of the compound in the extracts obtained from different methodologies.

**Table 2:** Amount of the compound (−)-mammea A/BB in *Calophyllum brasiliense* leaves extracts obtained by different extraction methods.

Extraction conditions	% (−)-mammea A/BB *
SFE	
10.92 MPa/313 K	5.6 ± 0.4 ^a^
24.41 MPa/333 K	4.7 ± 0.2 ^b^
25.00 MPa/313 K	5.3 ± 0.1 ^a,b^
Soxhlet	
Dichloromethane	0.3 ± 0.03 ^c^
*n*-Hexane	3.6 ± 0.2 ^d^

Means with the same letter are not significantly different from each other (Tukey’s test, P < 0.05). * % (−)-mammea A/BB = (mass of (−)-mammea A/BB/extracted mass) × 100.

Extracts obtained by SFE contained much higher concentrations of (−)-mammea A/BB compared to the organic solvent extraction, especially the extract obtained at 313 K and 10.92 MPa. These results indicate that, under the evaluated conditions, supercritical carbon dioxide proved to be a more efficient solvent in terms of (−)-mammea A/BB extraction selectivity. The results also suggested that the content of (−)-mammea A/BB increased with decreasing organic solvent polarity.

### 2.3. Total Phenols

Results for content of total phenols depend on the chemical nature and structure of the phenolic compounds in the extract. Since plants of the genus *Calophyllum* include compounds such as coumarins, biflavonoids and xanthones, it was expected that the content of total phenols would be quite expressive. Table 3 shows quantities of total phenols as milligrams of gallic acid equivalent (GAE) per gram of extract obtained. The data shows that the extract obtained with dichloromethane furnished the highest content of phenols expressed as GAE. The hexane extract exhibited less significant amounts, which is expected because of its low solvent polarity. In the case of SFE, the extract obtained under 25.00 MPa and 313 K conditions provided a higher content than the others. Results clearly indicate a relationship between the amount of phenols and pressure used. In an isotherm, the raise in pressure is proportional to the increase in the quantity of total phenols. For nearly similar pressures we can note that an increase in temperature decreases the total phenols content. This fact can be explained due to the fact that these compounds are heat sensitive, or due to the presence of degradation phenomena.

**Table 3:** Total phenols (GAE) content for supercritical CO_2_ and Soxhlet extractions.

Extraction condition	Mean Absorbance	Total phenols (mg of GAE/g of extract)
SFE		
10.92 MPa/313 K	0.126 ± 0.01	15.06 ± 1.75
24.41 MPa/333 K	0.230 ± 0.02	26.98 ± 2.90
25.00MPa/313 K	0.285 ± 0.01	33.29 ± 1.75
Soxhlet		
Dichloromethane	0.360 ± 0.01	41.89 ± 1.75
*n*-Hexane	0.159 ± 0.01	18.84 ± 1.75

### 2.4. Antioxidant Activity- DPPH Method

Antioxidant activities detected in complex systems, such as vegetal materials, may be caused by several classes of components as well as by synergic effects or interactions that occur between them. According to this fact, a specific antioxidant activity is totally dependent on the corresponding extract’s composition. Table 4 shows the mean percentage antioxidant activity and IC_50_ observed for the different extracts concentrations by the DPPH method.

**Table 4:** Antioxidant activity data (%) obtained for SFE and Soxhlet extracts by the DPPH method.

	Antioxidant Acitivity Percentage (AA%)						IC_50_
	Concentration of the extracts (µg/mL)						(µg/mL)
Extraction conditions	25	33.33	50	150	250	350	
SFE							
10.92 MPa/313 K	15.40	18.48	31.22	50.12	69.03	79.54	149.35 ^a^
24.41 MPa/333 K	12.44	17.30	21.59	50.09	66.08	78.08	149.13 ^a^
25.00MPa/313 K	13.60	17.29	28.95	54.91	68.17	76.18	131.73 ^a^
Soxhlet							
Dichloromethane	3.94	9.00	20.66	40.41	58.52	75.60	206.58 ^b^
*n*-Hexane	8.02	9.39	18.21	39.90	52.34	60.46	242.84 ^b^

Means with the same letter are not significantly different from each other (Tukey’s test, P < 0.05).

Extracts obtained by the Soxhlet method presented higher IC50 values than those obtained by SFE, which means lesser antioxidant activity. Both extracts (hexane and dichloromethane) also presented minor amounts of mammea A/BB, a compound already recognized in the literature for its antioxidant activity. On the contrary, the SFE extracts exhibited higher amounts of this constituent (Table 2), and, correspondently, the best antioxidant activity, so it can be suggested that this data is probably correlated to this result. According to Reynertson *et al.* [34], an extremely active extract has an IC50 value lower than 50 μg/mL, so it can be attributed a moderate activity for SFE *C. brasiliense* extracts.

### 2.5. Mathematical Modeling

Extraction modeling is relevant for the optimization of natural product extraction projects such as the definition of the extractor’s volume and for the prediction of extraction behavior throughout the process (total time of extraction for a specific set of operation conditions). Table 5 shows the parameters values used in kinetic extraction model and estimated values of constant k corresponding to each run. It can be seen that the leaves extracts are more easily extracted at 24.41 MPa and 333 K. Parameters k are lower and very close to each other under the other conditions. In the case of extractions of *C. brasiliense* leaf extract, when the temperature is constant, this parameter is affected by pressure. In fact, it is bigger for the 333 K isotherm, where the pressure rose from 17.67 MPa to 24.41 MPa the value of parameter k increased from 3.73 to 6.12 cm³/g min. On the other hand, for the 313 K isotherm, a pressure increase caused a small decrease in the values of parameter k. 

**Table 5:** Parameters of mass transfer and constant k for the mathematical modeling according to extraction conditions.

Parameters	Extraction condition				
	P = 10.92 MPa	P = 15.00 MPa	P = 17.67 MPa	P = 24.41 MPa	P = 25.00 MPa
	T = 313 K	T = 313 K	T = 333 K	T = 333 K	T = 313 K
Q_f_ (mL/min)	3	3	3	3	3
m (g)	20.0681	20.0057	20.0136	20.0035	20.0028
ε	0.87	0.85	0.85	0.85	0.85
ρ_CO2_ (g/mL)	0.6813	0.7811	0.6813	0.7811	0.8802
ρ_bed_ (g/mL)	0.1169	0.1165	0.1166	0.1165	0.1165
u (cm/min)	0.5405	0.5533	0.5533	0.5533	0.5533
C_eq_ (g/mL)	7.768 × 10^−4^	1.271 × 10^−3^	1.385 × 10^−3^	2.007 × 10^−3^	2.739 × 10^−3^
k (mL/g min)	3.59	3.27	3.73	6.12	3.19

**Figure 3:**
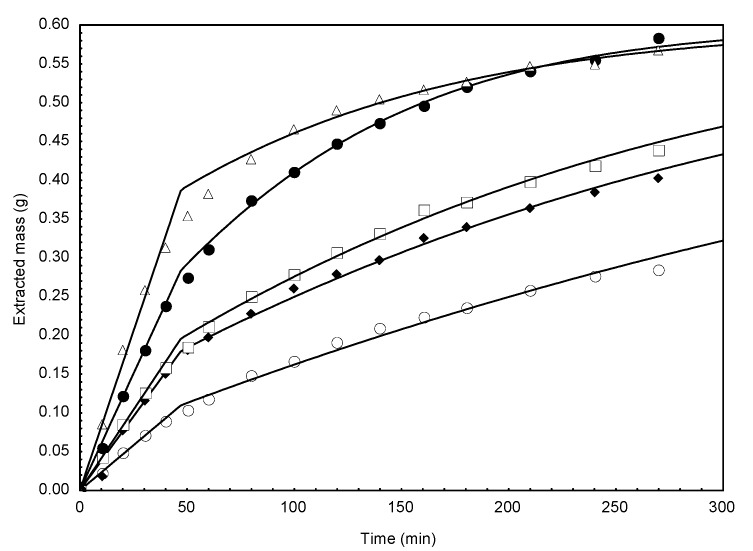
Experimental and calculated extraction for 30-mesh leaves of *C. brasiliense*. (○) P = 10.92 MPa, T = 313 K; (♦) P = 15.00 MPa, T = 313 K; (□) P = 17.67 MPa, T = 333 K; (●) P = 24.41 MPa, T = 333 K; (∆) P = 25.00 MPa, T = 313 K; (─) mathematical model.

Figure 3 shows experimental and modeled extraction kinetics of compounds for *C. brasiliense* leaves.

It can be observed from Figure 3 that the model adequately represents the extraction kinetics for all investigated conditions. 

## 3. Experimental

### 3.1. Pre-treatment of the Vegetal Matrix

The leaves of *Calophyllum brasiliense C*ambess were collected on Cardoso Island in the state of São Paulo, Brazil, in December 2010, and the exsiccate was deposited in the Herbarium of the Botanic Institute of São Paulo as number SP363818. The botanical material was dried in a circulating air oven (Quimis Q-31) at 313 K temperature. After 72 h, the leaves were milled in a home processor (WALITA RI7625). Tyler sieves (W. S. Tyler, Mentor, OH, USA) were used to classify the samples according to particle size. The leaves trapped in the 30 and 50 mesh sieves were chosen for further extraction steps.

### 3.2. Extraction- Organic Solvent Extraction

The organic solvent extraction was performed for 300 min according to the Adolfo Lutz Institute methodology (Instituto Adolf Lutz, [35]), using a Soxhlet apparatus. Dichloromethane (Nuclear, 99.6% purity) and hexane (Nuclear, 99.6% purity) were used due to their differences in terms of polarity and dielectric constant. For hexane and dichloromethane, the boiling points are 342 K and 313 K and the dielectric constants 1.88 and 8.93, respectively. The yields obtained for each solvent extraction were expressed and calculated in relation to the initial dry weight sample.

### 3.3. Extraction- Supercritical Fluid Extraction (SFE)

**Figure 4:**
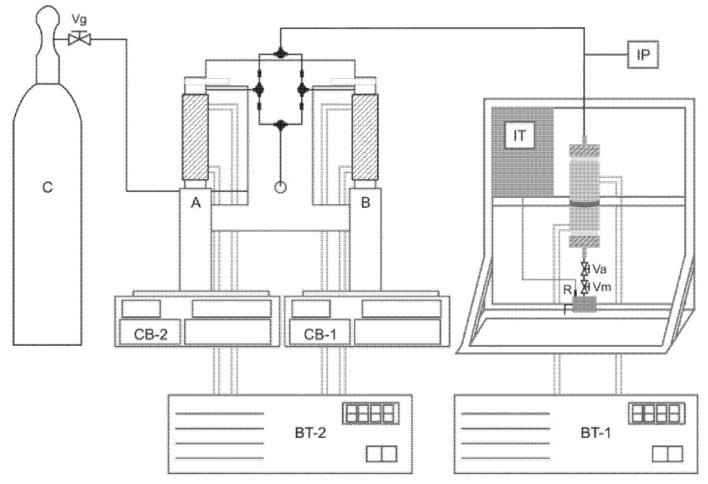
A and B –syringe pump; BT-1 and BT-2- thermostatic bath; C-CO2 cylinder; CB-1 and CB-2 –controller of syringe pump; IP –pressure indicator; IT –temperature indicator (thermal regulator); Va –needle valve; Vg –sphere valve; Vm –micrometering valve; R –collection reservoir.

SFE experiments were performed on a bench scale unit, as shown in Figure 4. The experimental model consisted of a CO_2_ cylinder (Figure 4 – C) (Air Liquide Brasil Ltda., 95% purity), two syringe pumps (Figure 4 – A and B) (Teledyne Isco, Model 500D), two thermostatic baths (Figure 4 – BT-1 and BT-2) (Quimis, Model Q214M2 and Tecnal, ModelTE-184), and one extractor with internal volume of approximately 170 mL (base diameter 2.85 cm and height 26.1 cm).

Approximately 20 g of *Calophyllum brasiliense* leaves were used in each experiment. They were previously dried, milled, sieved and placed in the stainless steel extractor; the remainder of the extraction cell was filled with glass spheres as an inert bed. Carbon dioxide, fed at the upper part of the extractor, passed through the inert bed and then on to the vegetal matrix. At the exit of the extractor, the extract was separated from the solvent by depressurization and the extracted mass was collected in an amber flask. The extraction was then performed up to 270 minutes for leaves. Runs were performed in triplicate for all conditions. The yield values presented in this work refer to the average yield.

Table 6 shows the operational conditions details. According to the literature, pressures between 15 and 40 MPa are more commonly used for phenolic compounds extraction [36]. Since temperature may affect the thermal stability of the solute and the characteristics of the matrix, and, occasioning, mass transference limitation, mild extraction conditions ranging between 313 and 333 K are often employed for the extraction of medicinal herb compounds [37].

**Table 6:** Parameters used for the supercritical fluid extraction experiments.

Pressure (MPa)	Temperature (k)	Density (g/mL)	Flow (mL/min)
10.92	313	0.6813	3
15.00	313	0.7811	3
17.67	333	0.6813	3
24.41	333	0.7811	3
25.00	313	0.8802	3

### 3.4. Quantification of (−)-Mammea A/BB

The quantification of (-) mammea A/BB in the extracts was based on the methodology described by Brenzan *et al*. [38] using a High Performance Liquid Chromatography (HPLC) device. The equipment consisted of a Varian 920 LC with a DAD (diode array) detector, equipped with a quaternary pump and auto sampler injector, controlled by Galaxie Software, reverse phase column Metasil ODS 150 × 4.6 mm with a 5 μm particle (METACHEM), and a column temperature controlled at 303 K.

The qualitative and quantitative analysis were performed by using a gradient elution protocol constituted by acetonitrile (J.T. Baker, 99.99% purity)-water as mobile phase in the following proportions: 5:95 to 55:45 v/v (0–10 min.), 55:45 to 80:20 v/v (10–20 min.), 80:20 to 100:0 v/v (20–30 min.) and 100% acetonitrile (30–40 min.), with a flow rate of 0.6 mL/ min.

The calibration curve was established by the external standard method using (−)-mammea A/BB coumarin isolated from the leaves of *Calophyllum brasiliense*, according to Brenzan *et al.* [19]. All measurements were undertaken in triplicate.

### 3.5. Total Phenol Contents

To determine the total phenolic contents, the method described by Meda *et al*. [39] was employed with modifications, using the Folin-Denis (Sigma-Aldrich, 100% purity) instead of the Folin-Ciocalteau reagent. The color of the solution is expected to change from green to blue in positive reactions.

Extracts were prepared at a concentration of 1 mg/mL in methanol (FMaia, 99.8% purity). Next, 2.5 mL of 10% Folin-Denis reagent solution (10 mL of the reagent in 100 mL of ultra-pure water) was added in a 0.5 mL extract solution. Finally, 2.0 mL of 14% sodium carbonate solution (Nuclear, 99.9% purity, 14 g of the reagent in 100 mL of ultra-pure water) was added after 5 minutes. The mixture was kept in the dark for 2 h. The absorbance was measured at 760 nm in a spectrophotometer (Shimadzu, UV-1203). For the negative control, a mixture of 0.5 mL methanol, 2.5 mL of 10% Folin-Denis reagent and 2.0 mL of sodium carbonate solution was used.

Gallic acid (Vetec), recognized as an antioxidant agent, was used as standard to construct the calibration curve. Concentrations ranging from 0.8 µg/mL to 7 µg/mL were applied and the preparation of these solutions followed the description above. The total phenolic contents was determined by the intersection of the absorbance of the samples across the calibration curve (R^2^ = 0.9991). Total phenolic content was expressed as mg of gallic acid equivalents (GAE) per g of extract.

### 3.6. Antioxidant Activity

The antioxidant activity of the extracts was evaluated according to the methodology proposed by Blois [40] and Brand-Williams *et al*. [41]. This method measures the sequestering activity of the free radical 2,2-diphenyl-1-picryl hydrazyl (DPPH^●^), purple colored, since it is reduced by antioxidant molecules forming yellow colored diphenylpicryl hydrazine.

The extracts were diluted in methanol up to concentrations that varied from 25 to 350 μg/mL. Next, 2,850 μL of the DPPH solution (0.6 mM) were added to150 μL of each tested sample. For the blank control, the volume of the samples was substituted by distilled water. The reaction was kept for 1 h at room temperature, in the dark, and the absorbance was measured at 515 nm.

The antioxidant activity (AA%) is expressed as a percentage of DPPH radical elimination, calculated according to the following equation:

AA% = [(1 – A.sample)/A.blank] × 100

Where A.blank is the absorbance of the blank and A.sample is the absorbance of the extract solution. The concentration of the extracts resulting in 50% of inhibition (IC_50_) was calculated from the inhibition percentage plotting graph. All tests were run in triplicate, and the average value was calculated.

### 3.7. Mathematical Modeling

The kinetic curves using CO_2_ extraction of *C. brasiliense* were modeled using a second-order empirical model proposed by Corso *et al*. [42] and De Souza *et al*. [43], that does not require knowledge of the axial concentration profile of the desired chemical species throughout the extraction bed. The equation of mass balance of concentration of extract in the fluid phase results in the following differential equation:

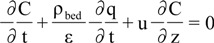

where C is the concentration of extract in the solvent (g/mL), q is the bioactive compounds concentration in the solid matrix (g_extract_/g_solid_), ρ_bed_ is the density in the bed (g/mL), u is the interstitial velocity (cm/min), t is the extraction time (min), ε is the bed porosity, z is the coordinate in the axial direction of the bed (cm). The next equation assumes that the extraction rate is proportional to the product of extraction capacity of the solvent in fluid phase (C_eq._ –C) and the oil concentration in solid matrix (q):

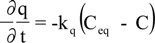

in which, k is the kinetic constant (mL/g min), C_eq_ is the equilibrium concentration of extract in the solvent (g/mL). 

This equation represents the analytical solution of model:

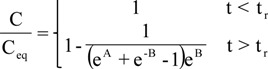

where t_r_ = L/u is the residence time (min), L is the length (cm) of column, A = (z/u)β, B = (-tu + z)β/αu, β = kc_eq_α and α = ρ_bed_q_0_/εC_eq_.

The extracted mass as a function of time was calculated by the equation:

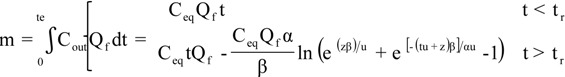

in which Q_f_ is the flow rate of the solvent and C_out_ is the concentration of extract in the fluid phase at the extractor outlet. Constant k was determined by minimizing the target function defined by the equation:

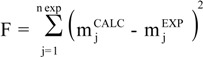

in which 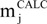 is the calculated extracted mass, 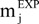 is the mass experimentally obtained, n exp is the number of experimental data of the kinetic curve.

## 4. Conclusions

*Calophyllum brasiliense* leaves extracts obtained by supercritical extraction presented lower total yields but higher quantities of the compound (−)-mammea A/BB when compared to the extracts obtained by the Soxhlet method. For pressures lower than 19 MPa, an increase in temperature from 313 K to 333 K decreased the overall yield. The SFE method used for the extraction of (−)-mammea A/BB proved to be more promising than conventional methods used in the extraction and purification of *C. brasiliense* extracts. Almost all extracts presented considerable phenolic compound values, which can be attributed to the chemical composition of *Calophyllum brasiliense*, reported to have xanthones, coumarins and biflavonoids in its composition. The higher antioxidant activity established by the DPPH method was assigned to the extracts obtained by SFE. A second-order kinetic model adequately represented the experimental extraction kinetic curves for all studied conditions with only one estimated parameter.
